# Comparative transcriptome analysis reveals a rapid response to phosphorus deficiency in a phosphorus-efficient rice genotype

**DOI:** 10.1038/s41598-022-13709-w

**Published:** 2022-06-08

**Authors:** M. Asaduzzaman Prodhan, Juan Pariasca-Tanaka, Yoshiaki Ueda, Patrick E. Hayes, Matthias Wissuwa

**Affiliations:** 1 Crop, Livestock and Environment Division, Japan International Research Center for Agricultural Sciences, Tsukuba, Ibaraki Japan; 2 School of Biological Sciences, The University of Western Australia, 35 Stirling Highway, Perth, WA 6009 Australia

**Keywords:** Transcriptomics, Abiotic, Plant molecular biology, Plant physiology, Plant stress responses

## Abstract

Phosphorus (P) is an essential plant nutrient. Most rice growing lands lack adequate P, requiring multiple P fertiliser applications to obtain expected yields. However, P fertiliser is environmentally damaging, and already unaffordable to the marginal farmers. This warrants developing P-efficient rice varieties that require less P to produce the expected yield. However, genetic factors underlying P-use efficiency (PUE) in rice remain elusive. Here, we conducted comparative transcriptome analysis using two rice varieties with contrasting PUE; a P-efficient landrace DJ123 and a P-inefficient modern cultivar IR64. We aimed to understand the transcriptomic responses in DJ123 that allow it to achieve a high PUE under low P conditions. Our results showed that both DJ123 and IR64 had replete tissue P concentrations after 48 h of P deprivation. Yet, DJ123 strongly responded to the external low P availability by inducing P starvation-inducible genes that included *SPX2*, *PHO1*, *PAPs* and *SQDs*, while these genes were not significantly induced in IR64. We envisage that the ability of DJ123 to rapidly respond to low P conditions might be the key to its high PUE. Our findings lay a valuable foundation in elucidating PUE mechanism in rice, thus will potentially contribute to developing P-efficient modern rice variety.

## Introduction

Phosphorus (P) is an essential macronutrient for plant growth, development, and yield. It is a critical component in various biochemical processes like photosynthesis, respiration, and lipid metabolism. It is also a pivotal part of nucleic acids and the energy currency of the cell, ATP^1^. Under normal physiological conditions, plants take up P from soil as inorganic phosphate (Pi), H_2_PO_4_^−^. Soil Pi concentration across the globe ranges from < 0.6 to 11 µM with an average 3 µM^2^. However, plants require a far greater concentration of Pi at the intracellular level, ranging from 5 to 20 mM^1,3,4^. Therefore, most soils need to be supplemented by external P for optimal plant growth and development.

About half of the world-wide rice growing soils are deficient in P^5^. As such, rice farming heavily relies on external P fertiliser applications for achieving desired yield^6^. This P fertiliser dependence is greater in the economically poor regions, which are characterised by upland and rainfed rice farms^7^. However, most farmers in these regions, where rice demand is generally higher, already cannot afford the fertiliser costs or do not have access to P fertilisers^7–9^. This situation is bound to be aggravated as the demand for rice production is on the rise to feed an alarmingly increasing world population^10,11^. Furthermore, the application of P-fertilisers in agricultural systems has already caused some profound concerns. For example, depletion of rock phosphate reserve within next 50–100 years^12,13^ that is used as a raw material for manufacturing P fertiliser^14,15^ and negative environmental impacts from excess fertiliser application, including eutrophication^16^. This situation warrants developing rice varieties that can thrive on low P soils, with minimal fertiliser and still achieve the desired yield^17,18^.

There is a world-wide effort to breed P-efficient rice cultivars^19^. Rice breeders cross modern high yielding varieties with P-efficient rice varieties, which are generally lower-yielding; to effectively transfer P-efficient traits to the modern varieties^20^. The P-efficient rice breeding programs generally aim for two traits, namely (1) P acquisition efficiency (PAE) that accounts for greater P uptake from soil and (2i) P utilisation efficiency (PUE) that produces greater yield and biomass per unit P^21^.

DJ123 and IR64 are two cultivars contrasting in P use efficiency^22^ that are often used in rice breeding programs as a donor and recipient of P-efficiency traits, respectively. DJ123 is an *aus*-type rice variety that thrives in low P soil^23,24^ by operating at a greater PAE^24,25^ and PUE^22,26^. On the other hand, IR64 is a popular high-yielding *indica* variety^27^ but poorly performs in low-P conditions^23^. However, the basic question remains to be elucidated as to how DJ123 differs from other genotypes that constitutes its remarkable tolerance to low P. This knowledge will likely provide the breeders with specific targets in P-efficient rice breeding.

Here, we aimed to carry out a comparative transcriptomics analysis between DJ123 and IR64 as it provides an wholistic approach for dissecting genotypic differences^28,29^. Our goal was to gain insights on the earlier events that potentially lead to low-P tolerance in DJ123. In accordance, we designed the experiment in a way that allowed us to capture the immediate transcriptomic responses to low P (see “Materials and methods” section). We included IR64 as a contrasting genotype. Then, we followed the genotypic differences in the transcriptomes through the differential gene expression analysis.

## Results

Our experimental design (Supplementary Fig. S1) attempted to distinguish long-term from short-term responses to P starvation. The long-term response is expected to be captured in the contrast between the HPC and LPC treatments, which supplied either high P continuously (HPC) or low P continuously (LPC) throughout the experimental period. Short-term P starvation was induced in both high and low-P treatments by stopping P supply 48 h prior to harvesting the plants and labelled as HPS and LPS, respectively. Thus, plants in the HPS treatment were supplied with P in excess of demand for 40 days and should have maintained tissue P concentrations well above deficiency levels but were expected to have reacted to a shift to a P-free growth medium 48 h prior to sampling. We adopted this transfer treatment approach to ensure that plants were exposed to only short-term external P deficiency, thus allowing us to capture the genotypic differences in short-term responses to P starvation.

### Genotypic variation in P-dependent growth and tissue P concentrations

**Figure 1:**
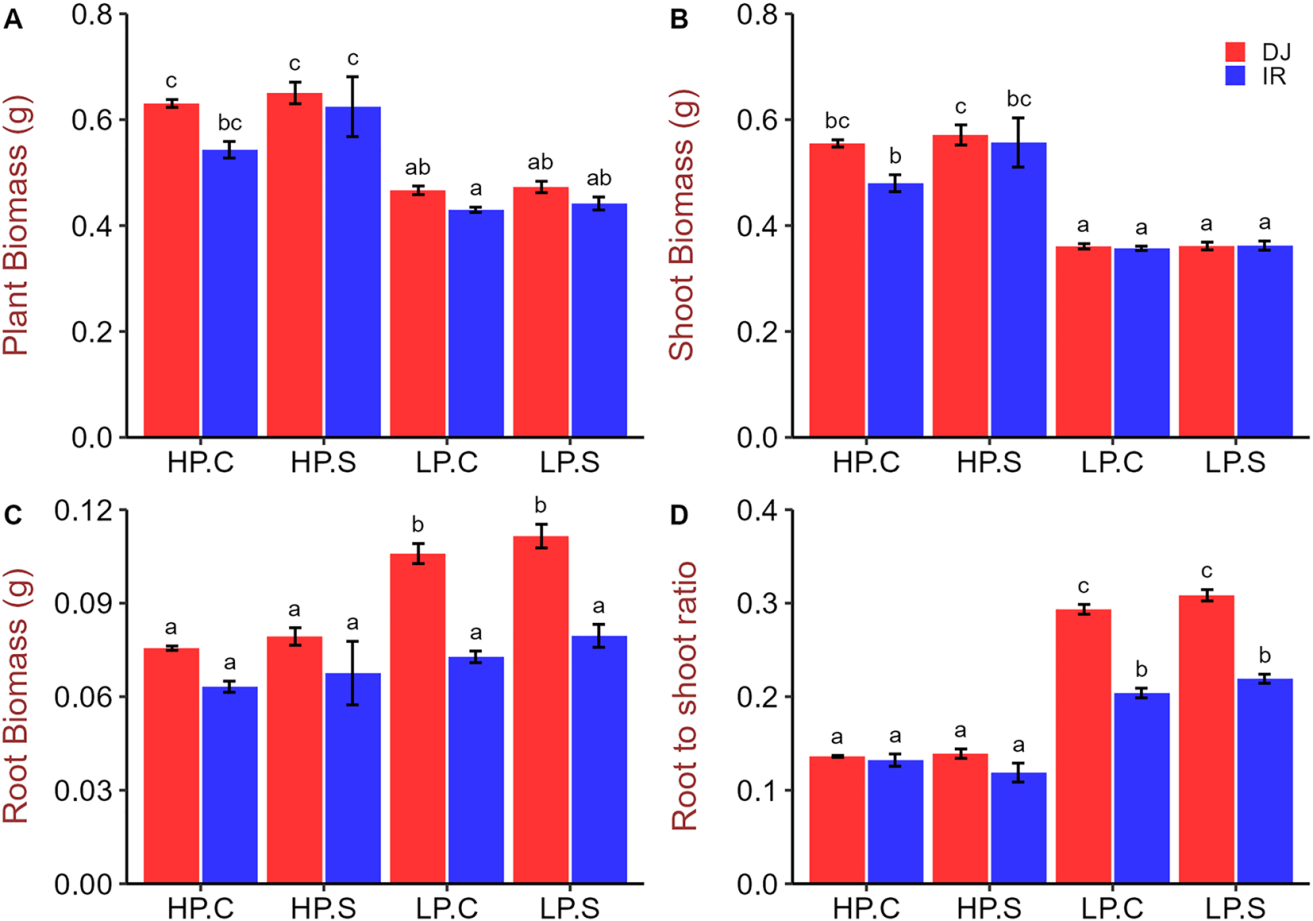
Whole plant, shoot and root biomass production and root-to-shoot biomass ratio of DJ123 and IR64 grown under varying phosphorus supply. Data are the mean ± SE of three to four independent replicates. ‘HP’ and ‘LP’ stand for high- and low-phosphorus supply, respectively. Plants were grown on HP and LP treatments for 40 days after germination. Then, both groups were splitted into two sub-groups. P supply was continued as usual for one sub-group of each treatment (labelled as ‘C’) and stopped for the other group (labelled as ‘S’). After approximately 24 and 48 h, LP and HP sub-groups were harvested, respectively. Significant differences among treatments were determined by ‘generalised least square’ model, separated by Tukey’s test (P < 0.05), and are indicated by different letters.

Low P supply over 40 days after germination reduced shoot biomass by around 65–70% in both DJ123 and IR64 (Fig. 1A,B) but had the opposite effect on root biomass, which significantly increased (140%) in genotype DJ123 relative to the high P treatments. Root biomass of IR64 remained non-responsive to P deficiency (Fig. 1C). Both genotypes increased root-to-shoot biomass ratio under LPC compared to that in HPC (Fig. 1D). However, this increase was twice as pronounced in DJ123 because of its significantly higher root biomass in both low-P treatments (Fig. 1D).

**Figure 2:**
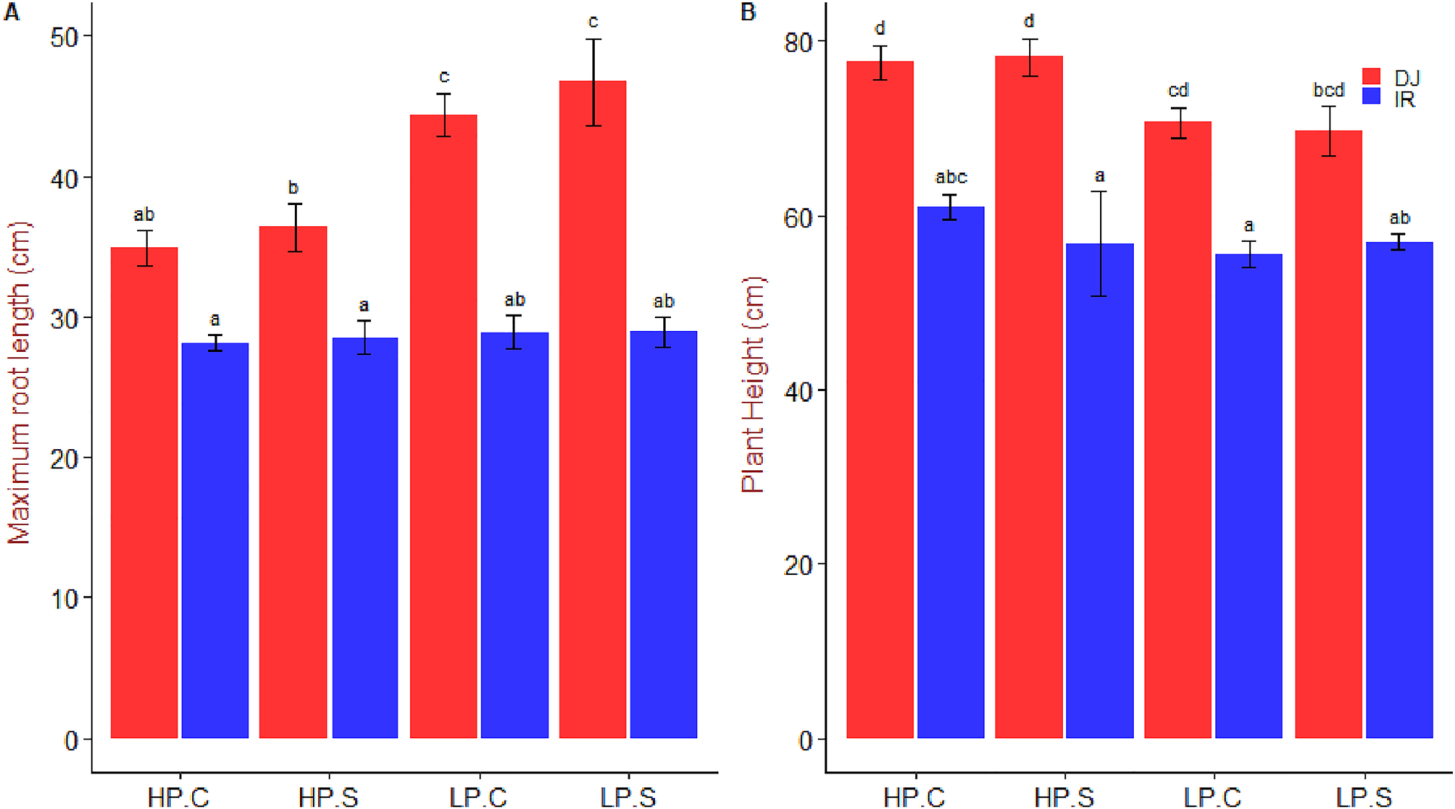
Maximum root length and plant height of DJ123 and IR64 under varying phosphorus supply. Data are the mean ± SE of four independent replicates. ‘HP’ and ‘LP’ stand for high- and low-phosphorus supply, respectively. Plants were grown on HP and LP treatments for 40 days after germination. Then, both groups were splitted into two sub-groups. Phosphorus supply was continued as usual for one sub-group of each treatment (labelled as ‘C’) and stopped for the other group (labelled as ‘S’). After approximately 24 and 48 h, LP and HP sub-groups were harvested, respectively. Significant differences among treatments were determined by ‘generalised least square’ model, separated by Tukey’s test (P < 0.05), and are indicated by different letters.

Maximum root length increased in DJ123 in response to low availability of P but remained constant in IR64 (Fig. 2A). DJ123 was consistently taller compared to IR64 but this difference in plant height decreased under P deficiency (Fig. 2B).

**Figure 3:**
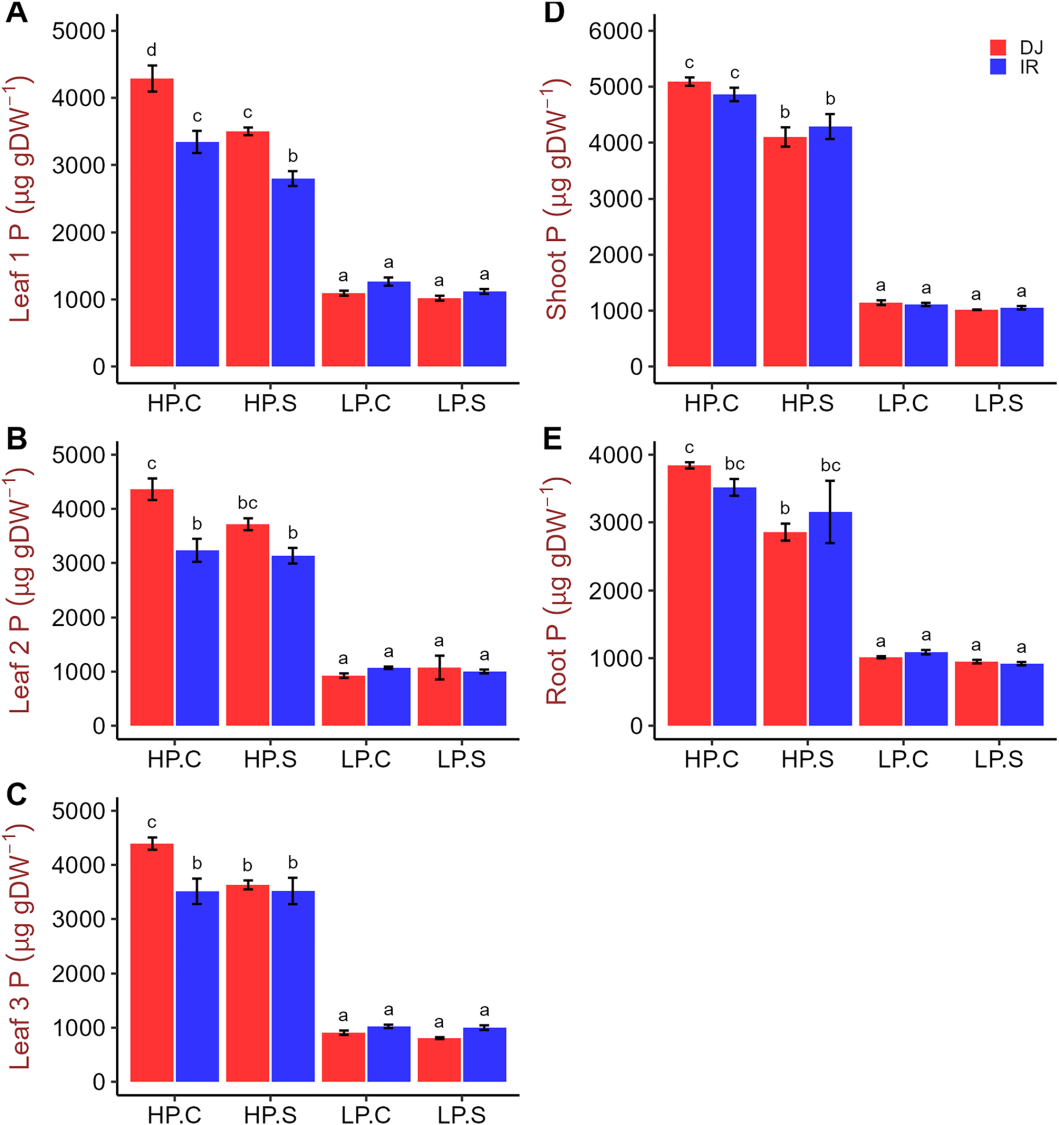
Phosphorus concentration in different tissues of DJ123 and IR64 grown under varying phosphorus supply. Data are the mean ± SE of four independent replicates. ‘HP’ and ‘LP’ stand for high- and low-phosphorus supply, respectively. Plants were grown on HP and LP treatments for 40 days after germination. Then, both groups were splitted into two sub-groups. Phosphorus supply was continued as usual for one sub-group of each treatment (labelled as ‘C’) and stopped for the other group (labelled as ‘S’). After approximately 24 and 48 h, LP and HP sub-groups were harvested, respectively. Significant differences among treatments were determined by ‘generalised least square’ model, separated by Tukey’s test (P < 0.05), and are indicated by different letters.

**Figure 4:**
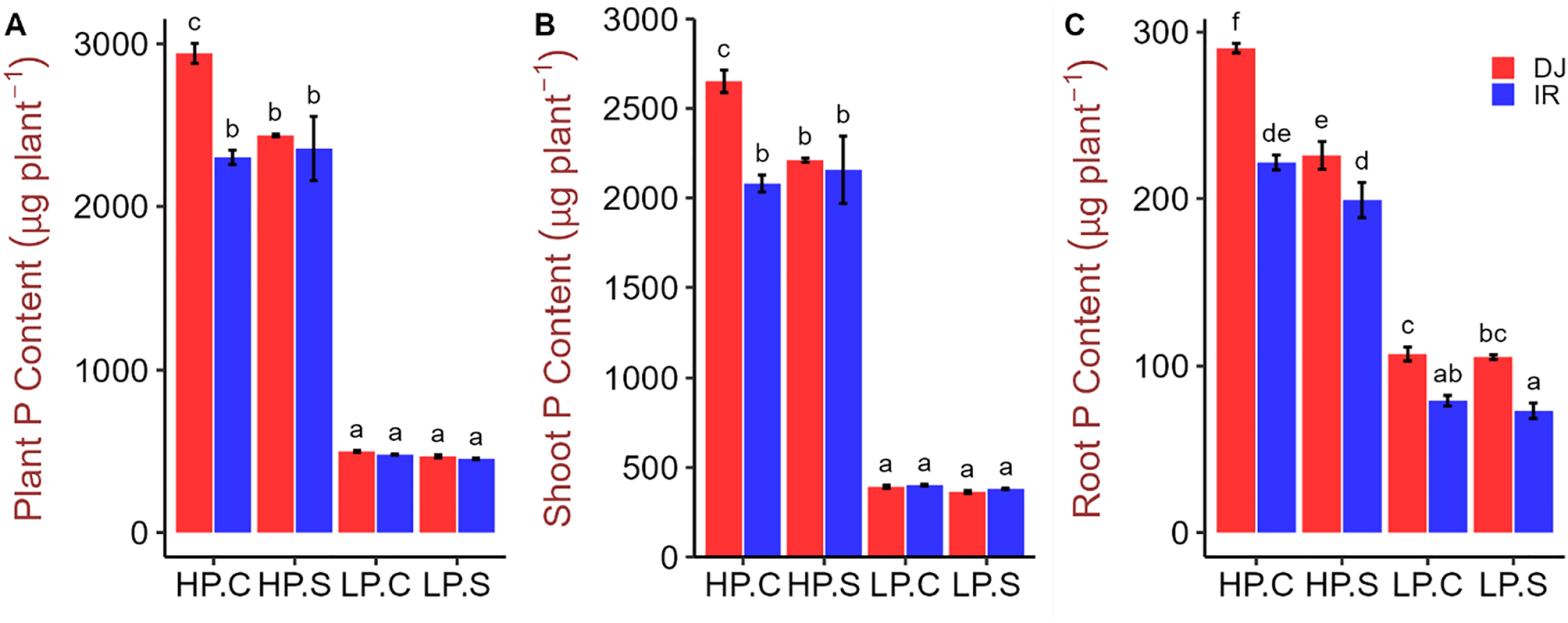
Phosphorus content in whole plant, shoot and root of DJ123 and IR64 grown under varying phosphorus supply. Data are the mean ± SE of four independent replicates. ‘HP’ and ‘LP’ stand for high- and low-phosphorus supply, respectively. Plants were grown on HP and LP treatments for 40 days after germination. Then, both groups were splitted into two sub-groups. Phosphorus supply was continued as usual for one sub-group of each treatment (labelled as ‘C’) and stopped for the other group (labelled as ‘S’). After approximately 24 and 48 h, LP and HP sub-groups were harvested, respectively. Significant differences among treatments were determined by ‘generalised least square’ model, separated by Tukey’s test (P < 0.05), and are indicated by different letters.

In the high P treatments leaf P concentrations of the top three youngest leaves remained within or even slightly above optimum tissue concentration range of 2.0–4.0 mg g^−1^^30^ (Fig. 3A–C), whereas leaf P concentrations dropped far below this optimum range in the low P treatments. Tissue P concentrations did not differ between genotypes under P deficiency but DJ123 tended to have slightly higher P concentrations under high P supply (Fig. 3A–E). The total plant P content was at least fourfold higher in the high-P treatment and DJ123 took up significantly more P than IR64 in the HPC but not the HPS treatment (Fig. 4A–C). In the low-P treatments, genotypic differences for P uptake were not significant, which matched the intended experimental design of supplying each genotype with an equal (low) amount of P that should have been taken up entirely. A clear difference in the distribution of P between root and shoot was observed with DJ123 having allocated more P to roots compared to IR64 (Fig. 4C).

### Global gene expression analysis by RNA-sequencing

RNA-seq of leaf and root tissues emanated into an average of 40 million raw reads per sample (Table 1). A set of about 20 million adapter-free and high-quality reads were filtered for each sample and mapped to the rice reference genome (*Oryza sativa* L. ssp. *japonica* cv. Nipponbare)^31^. More than 80% filtered reads were mapped uniquely while approximately 4% mapped multiple times and 10% remained unmapped. The overall alignment of the reads to the reference genome was 95% (Table 1).

**Table 1:** RNA-seq data statistics.

Genotypes	Tissues	Treatments	Read count	Clean reads (%)	Uniquely mapped reads (%)	Multiple mapped reads (%)	Unmapped reads (%)	GC (%)	Q20 (%)	Q30 (%)	Overall alignment rate (%)
DJ123	Leaf 1	HP.C	45,342,776	22,145,600 (49)	19,737,619 (89)	692,593 (3)	1,715,389 (8)	+	99	96	97
		HP.S	37,724,623	18,226,862 (48)	15,609,074 (86)	635,644 (3)	1,982,144 (11)	52	98	95	96
		LP.C	37,769,141	18,235,247 (48)	15,841,598 (87)	558,942 (3)	1,834,707 (10)	52	98	95	96
		LP.S	43,318,468	21,044,449 (49)	18,322,939 (87)	751,268 (4)	1,970,242 (9)	51	98	95	96
	Leaf 3	HP.C	46,905,192	22,949,402 (49)	20,297,136 (88)	755,126 (3)	1,897,140 (8)	53	99	96	96
		HP.S	41,135,822	20,011,509 (49)	17,135,319 (86)	997,149 (5)	1,879,041 (9)	52	99	96	96
		LP.C	35,750,745	17,252,127 (48)	14,728,478 (85)	623,740 (4)	1,899,909 (11)	51	98	95	95
		LP.S	47,472,197	23,020,267 (48)	18,795,279 (82)	2,060,998 (9)	2,163,990 (9)	51	98	95	95
	Root	HP.C	36,185,432	17,429,525 (48)	14,797,135 (85)	676,385 (4)	1,956,005 (11)	51	98	95	95
		HP.S	42,636,855	20,656,674 (48)	17,578,525 (85)	658,616 (3)	2,419,533 (12)	51	98	95	94
		LP.C	35,973,313	17,333,125 (48)	14,450,427 (83)	678,661 (4)	2,204,037 (13)	51	98	95	94
		LP.S	44,360,445	21,517,201 (49)	17,845,313 (83)	915,036 (4)	2,756,851 (13)	52	98	96	94
IR64	Leaf 1	HP.C	38,821,737	18,741,405 (48)	16,408,446 (88)	491,146 (3)	1,841,813 (10)	53	98	95	97
		HP.S	44,065,887	21,321,500 (48)	18,449,955 (87)	568,454 (3)	2,303,091 (11)	53	98	95	96
		LP.C	35,434,531	17,077,314 (48)	14,878,955 (87)	660,003 (4)	1,538,357 (9)	52	98	95	96
		LP.S	45,412,257	22,039,126 (49)	19,053,221 (86)	822,106 (4)	2,163,799 (10)	52	98	95	96
	Leaf 3	HP.C	36,295,171	17,450,071 (48)	15,218,917 (87)	507,037 (3)	1,724,117 (10)	52	98	95	97
		HP.S	43,353,859	21,028,237 (49)	18,350,129 (87)	573,417 (3)	2,104,691 (10)	52	98	96	96
		LP.C	37,390,312	18,061,709 (48)	15,654,221 (87)	789,054 (4)	1,618,435 (9)	51	98	95	96
		LP.S	42,166,059	20,442,760 (48)	17,095,040 (84)	1,315,842 (6)	2,031,878 (10)	51	98	95	96
	Root	HP.C	41,393,772	20,030,857 (48)	17,213,146 (86)	661,028 (3)	2,156,683 (11)	52	98	95	94
		HP.S	42,554,533	20,656,198 (49)	17,544,534 (85)	709,906 (3)	2,401,758 (12)	52	98	96	95
		LP.C	36,753,686	17,834,222 (49)	15,046,801 (84)	676,375 (4)	2,111,046 (12)	52	98	95	94
		LP.S	39,652,681	19,250,552 (49)	15,490,549 (80)	1,186,202 (6)	2,573,801 (13)	52	98	96	94
Average			40,744,562	19,739,831 (48)	16,897,615 (86)	790,197 (4)	2,052,019 (10)	52	98	95	95
Min			35,434,531	17,077,314 (48)	14,450,427 (80)	491,146 (3)	1,538,357 (8)	51	98	95	94
Max			47,472,197	17,077,315 (49)	20,297,136 (89)	2,060,998 (9)	2,756,851 (13)	54	99	96	97

**Figure 5:**
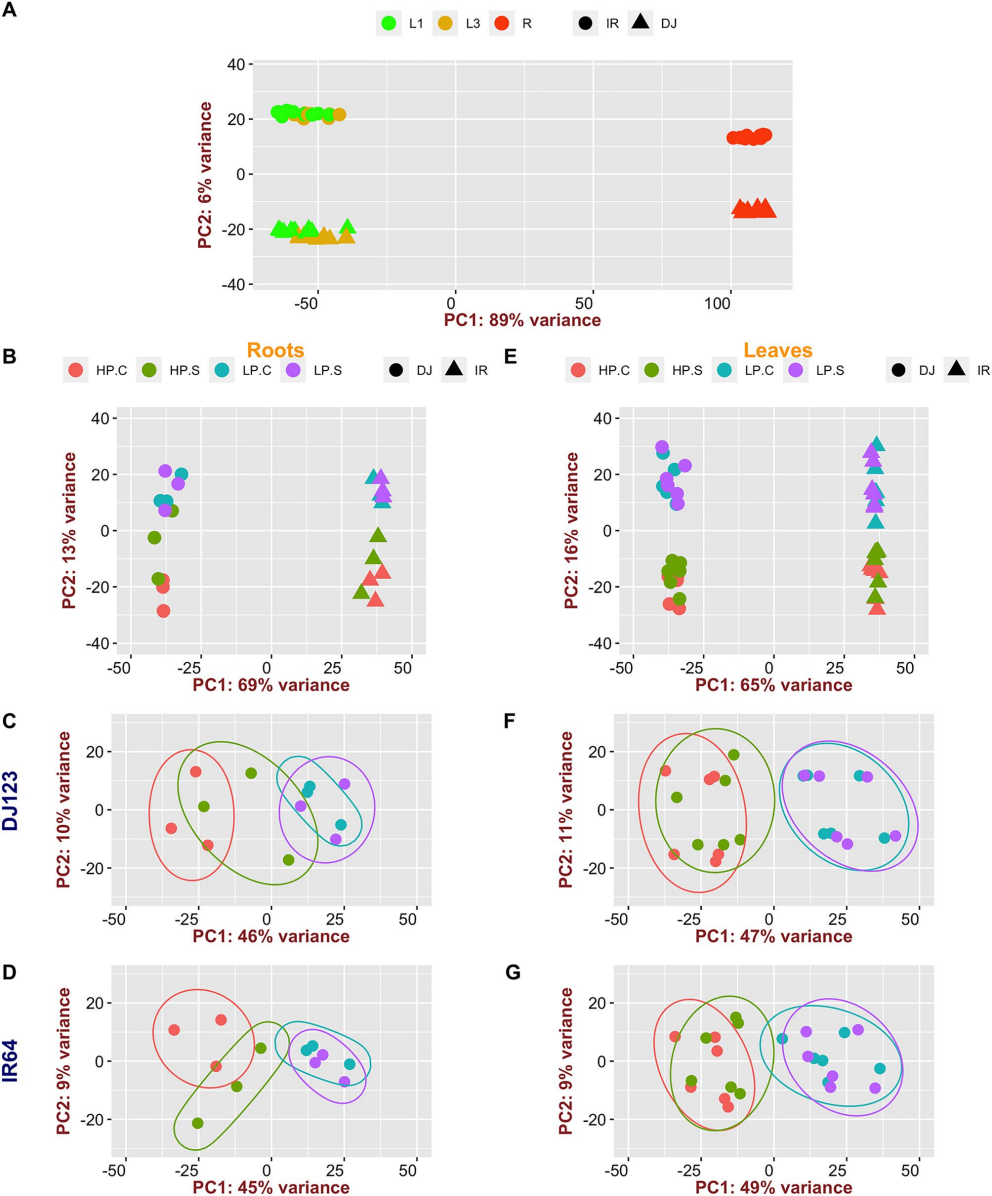
Principal component analysis of the RNA-seq results.

A first global analysis of the RNA-seq data through Principal Component Analysis (PCA) attributed 89% of the variance in the data to the difference between root and leaf tissues (Fig. 5A), indicating that these tissues should be analyzed separately. Subsequent separate analyses of root and shoot samples revealed that the factor genotype accounted for most of the variation whereas P treatment effects were less dominant (Fig. 5B,E). A further PCA analysis of root samples within an individual genotype suggested that different P supply treatments explained 46% and 45% of the variance in DJ123 and IR64, respectively (Fig. 5C,D). Very similar results were seen for the global gene expression patterns in leaf samples, where P treatments explained 47–49% (Fig. 5F,G) of the variance within each genotype. The main difference between expression patterns in root and shoot samples is the very clear separation by high *versus* low P supply in shoots (Fig. 5E,F), whereas this separation was less distinct in roots (Fig. 5B), especially in DJ123 (Fig. 5C) where HPS-treated roots appeared to be in between HPC and both low-P treatments. The shift of the HPS-treated root transcriptomes towards the LP-treated ones might suggest that the former has already responded to the 48 h of P deficiency in the growing medium, especially in DJ123. Based on these observations, we hypothesized that DJ123 responded faster to the short-term P deficiency (in high-P plants) than IR64, and in order to elucidate this fast response, we focused our further analyses on the HPC versus HPS comparison in root tissue.

### Differentially expressed genes (DEGs) by short-time deprivation of P in root samples

**Figure 6:**
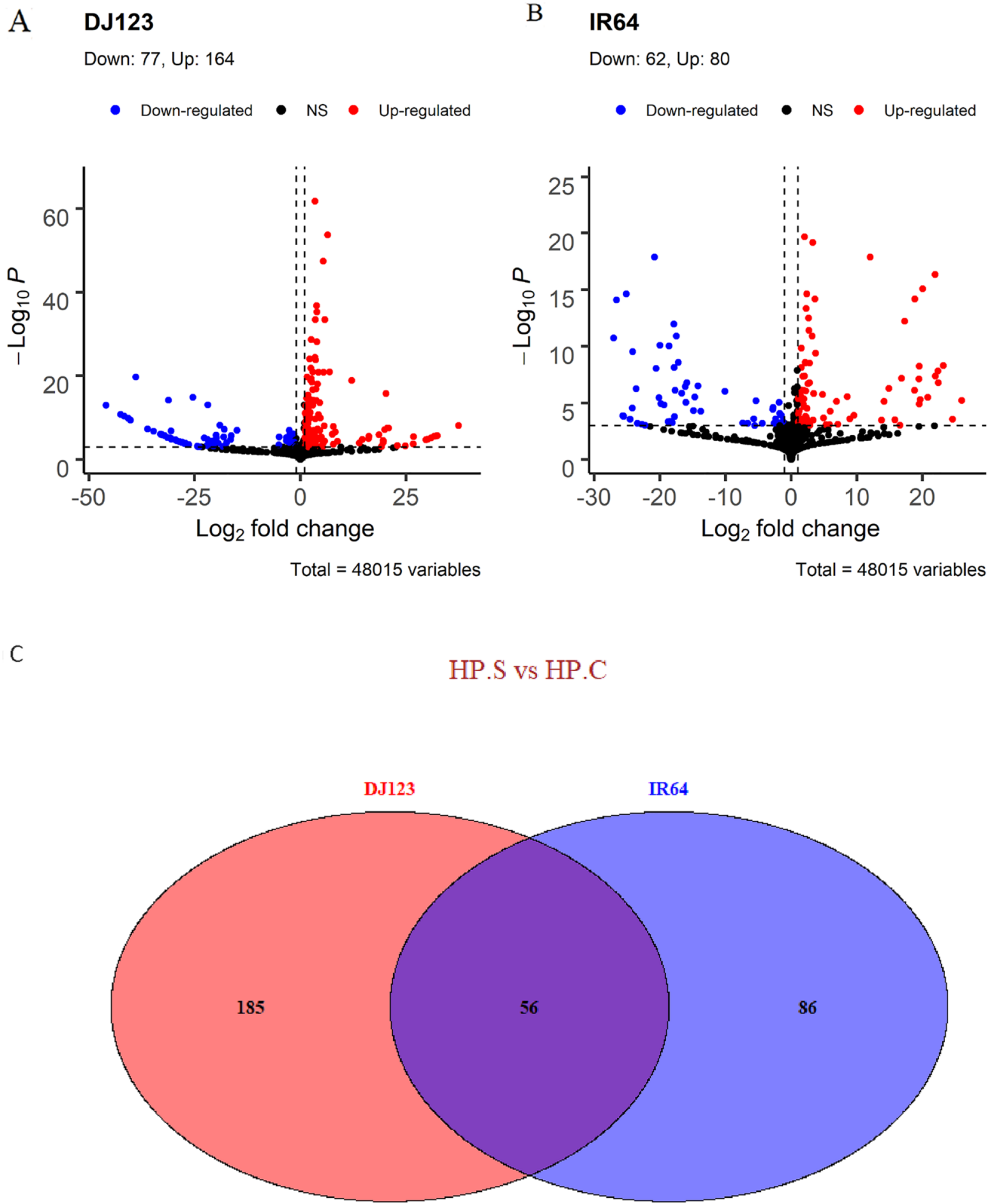
Volcano plots showing significantly up- or down-regulated genes in different tissues between DJ123 and IR64. The logarithm of fold change (log2) of each transcript is presented on x-axis, and the log10 of the P value are on y-axis. Thresholds are ‘pCutoff = 10e−16’ and ‘log2FoldChange > 1’. Venn diagram of the differentially expressed transcripts (DETs) in roots of DJ123 and IR64. The numbers of the DETs represent the DETs in HPS treatment against HPC treatment (HPS vs HPC).

Consistent with the PCA analysis, HPS-treated roots of DJ123 had more differentially expressed genes (DEGs) than those of IR64 and twice as many DEGs were upregulated in DJ123 compared to IR64 (Fig. 6A,B). Only 56 DEGs were commonly detected in both genotypes (Fig. 6C, Supplementary Table S1), thus most DEGs were genotype-specific, suggesting that IR64 and DJ123 have distinct response patterns to a short-term P deprivation.

### Gene Ontology analysis of the differentially expressed genes

**Figure 7:**
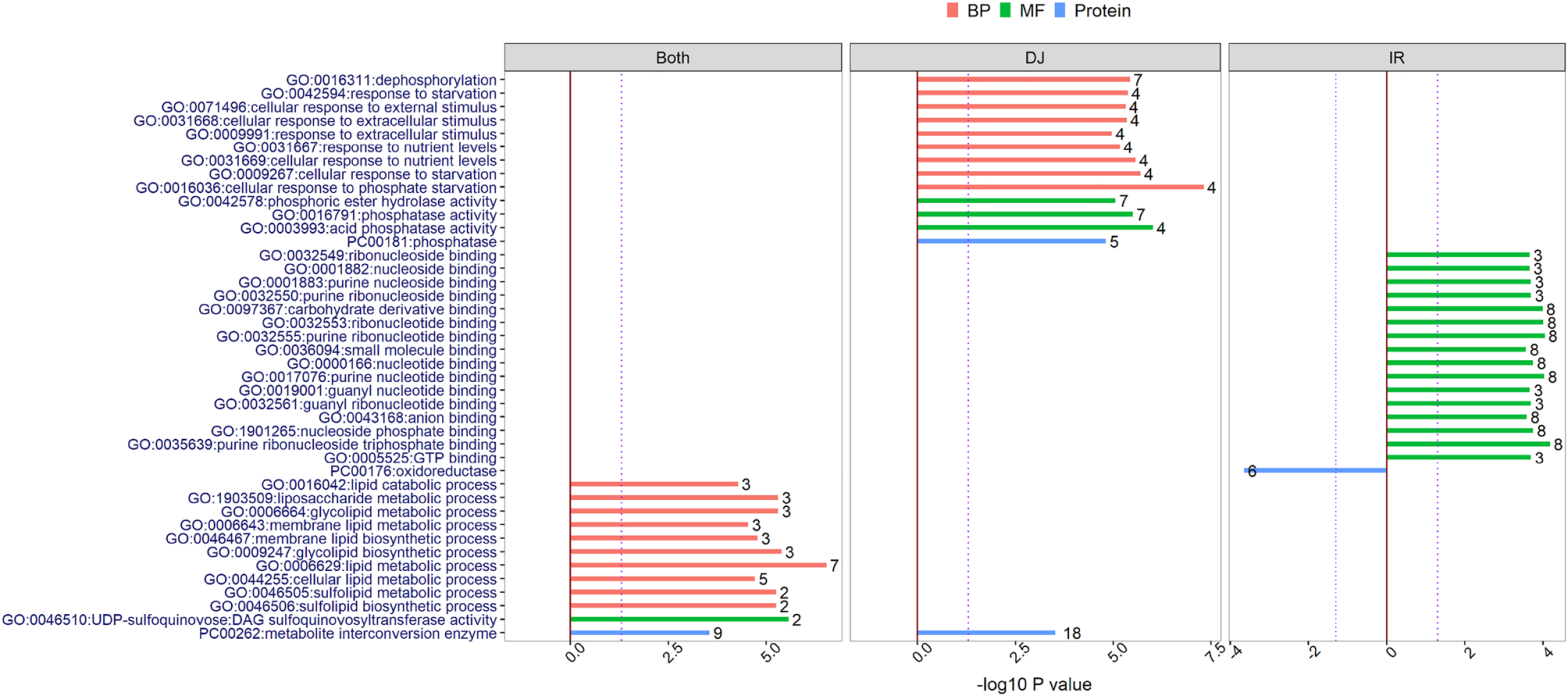
Gene Ontology (GO) enrichment analysis of differentially expressed genes (DEGs) in roots of rice seedlings. The Y-axis represents the enriched GO terms with their identification numbers. The X-axis represents the negative log10 of P values indicating the significance level of term-enrichment. The purple dotted-line represents P = 0.05. The up- and down-regulated genes under HPS treatment (compared to HPC treatment) were annotated separately for biological process (BP), molecular function (MF), cellular component, pathway and protein class (Protein) categories in PANTHER^32,33^. Different colors represent different categories. The categories that are absent in the graph were not significantly enriched. The bars on the right-hand side of (0,0) intercept indicate the GO-terms enriched by the up-regulated genes while the one on the left-hand side by the down-regulated genes. The numbers on top of the bars are the numbers of mapped genes. Three panels distinguish the GO-terms that were enriched in both genotypes (Both), only in DJ123 (DJ) and only in IR64 (IR).

Gene Ontology (GO) enrichment analysis was carried out to functionally annotate the HPS-induced DEGs that were DJ123- and IR64-specific as well as common in both genotypes. For each group, we analysed the up- and down-regulated genes separately for biological process (BP), molecular function (MF), cellular component, pathway and protein class (Protein) categories in PANTHER^32,33^. The GO-term enrichment analysis showed that DJ123 and IR64 had a common set of GO terms enriched as well as a genotype-specific set under HPS treatment (Fig. 7). The common GO terms were related with lipid metabolism and metabolite interconversion. DJ123-specific GO terms were related to responses to external stimuli, dephosphorylation and phosphatase activities. In addition, one GO term (PC00262:metabolite interconversion enzyme) from the common set of terms was further enriched by the DJ123 specific genes (Fig. 7). On the other hand, IR64-specific GO terms were all related to nucleotide binding. Interestingly, all the enriched GO terms were enriched by the up-regulated genes only except for the GO term ‘PC00176:oxidoreductase’, which was enriched by the IR64 down-regulated genes (Fig. 7). However, a single DEG was associated with multiple GO terms (Supplementary Table S1) as expected^32^. Accordingly, there were only 29 and 34 DEGs in the DJ123- and IR64-specific GO terms, respectively (Supplementary Table S2).

### Differentially expressed genes that enriched the GO terms under short-term P deprivation

**Figure 8:**
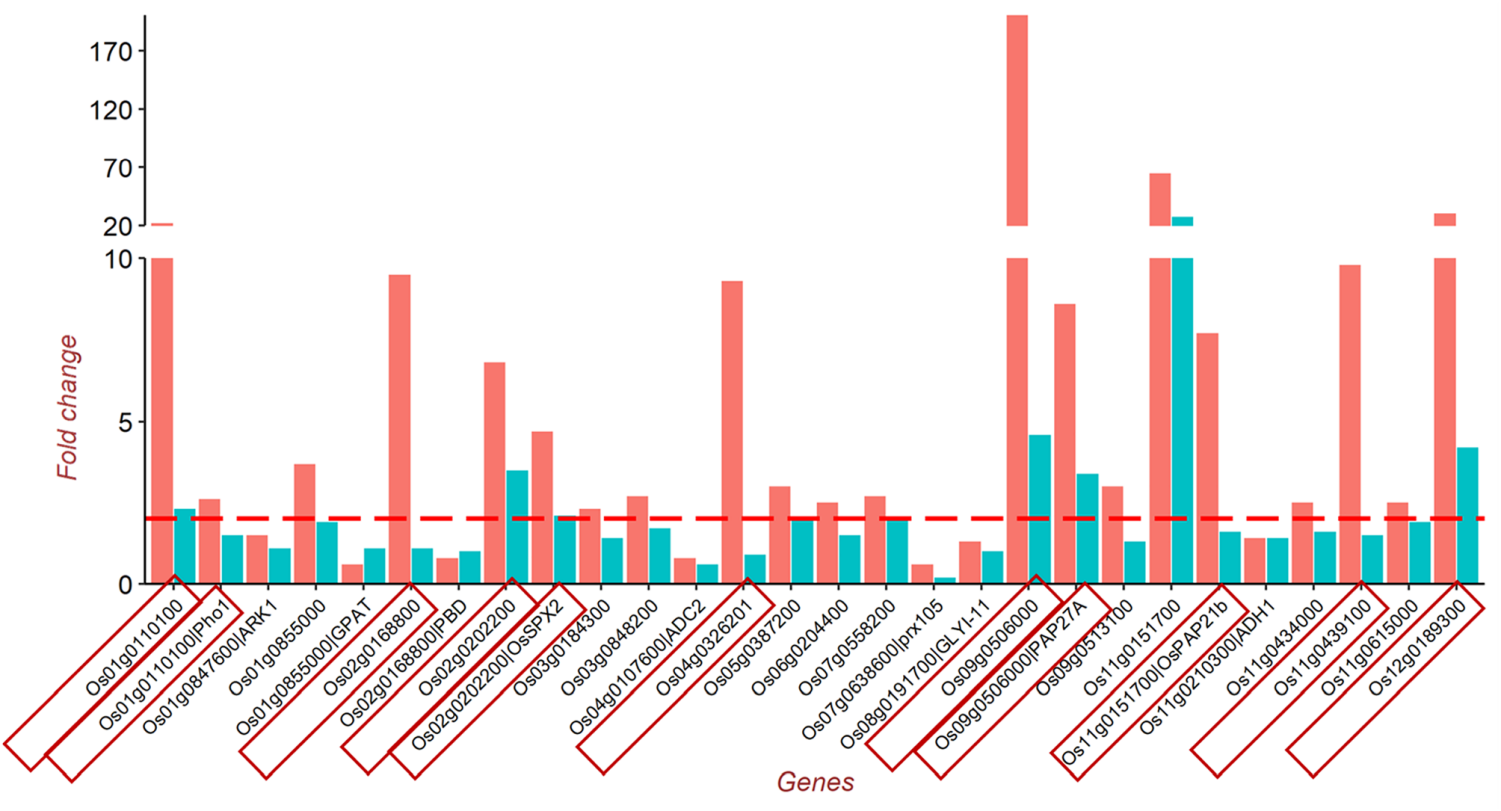
Fold change of the genes that enriched P-stress related GO terms in DJ123 under HPS treatment. Fold change was calculated by dividing the transcript abundance under HPS treatment by that under the HPC treatment in genotype-wise. The red line represents two-fold up-regulation of the corresponding gene in HPS compared to that in HPC. The red rectangular boxes highlight the genes, which are overdriven by DJ123 compared to IR64.

We hypothesized that the genes specifically induced in DJ123 by HPS treatment and/or molecular pathways enriched specifically in DJ123 are key components that characterize the response pattern of DJ123 to a short-term P deprivation. Accordingly, we compared the expression pattern of the genes from the enriched GO-terms (Supplementary TableS S1, S2) between DJ123 and IR64 genotypes under the HPS treatment (Fig. 8, Supplementary Fig S2). Considering together, our findings showed that the following genes were up-regulated to a greater extent in DJ123 compared to those in IR64 under the HPS treatment: *Os01g0110100* (*PHO1;1*), *Os02g0168800*, *Os02g0202200* (*SPX2*), *Os04g0326201*, *Os09g0506000* (*PAP27A*), *Os11g0151700* (*PAP21B*), *Os11g0439100* and *Os12g0189300* (Fig. 8); *PAP10a*, *SQD2.1* and *SQD2.2* (Supplementary Fig S2).

### Validation

To confirm the accuracy and reproducibility of the Illumina RNA-Seq results, we carried out qPCR assays for a panel of 6 differentially expressed genes under HPS: *SPX2, PHO1;1, PAP21b, PAP10a, SQD2.1, SQD2.2,* and two house-keeping genes *ELF1* and *UBI* (Supplementary Table S3). The ‘Fold change’ was calculated in the same way as in the transcriptome analysis, i.e., the transcript abundance under HPS treatment was divided by that under the HPC treatment in each genotype (Fig. 8). The expression pattern of the DEGs in the comparative transcriptome analysis under the HPS treatment (*SPX2, PHO1;1, PAP21b, PAP10a, SQD2.1 and SQD2.2*) (Fig. 8) corroborated with that in the qPCR assay (Supplementary Fig. S2). The level of expression of both house-keeping genes (*ELF1* and *UBI*) remained the same between DJ123 and IR64, as expected (Supplementary Fig. S2)*.*

## Discussion

### Characteristic response pattern of DJ123 to low P stress

DJ123 had a greater root biomass under low P treatments compared to that in high P, consistent with the increased maximum root length (Figs. 1, 2). In sharp contrast, root biomass, as well as maximum root length in IR64, remained non-responsive to the variation in P supply. Reflecting these contrasts, root to shoot ratio increased drastically in DJ123 by the low P treatment, indicating that DJ123 invested more resources to root growth than to shoot growth as compared with IR64. These results suggest that the low-P treatments in the experiment created P stress to the plants and triggered genotype-specific responses. Preferential resource allocation of DJ123 to root growth under P deficient conditions, as reported in previous studies^22,24,34^, is associated with its greater ability to explore larger soil volume, thus allowing efficient acquisition of scarcely available P^24^. In root, DJ123 significantly decreased P concentration by 48 h of P deprivation, while IR64 did not. Similar trend was observed for shoot and leaf 1 samples (Fig. 3). These observations show that DJ123 more drastically responds to low P stress than IR64, which could be a strategy to cope with low P stress more effectively.

### DJ123 induces P starvation responsive genes more strongly than IR64

Comparative transcriptomics have effectively been used to dissect underlying genetic mechanisms of a trait in rice^28,29^. To reveal genetic components that characterizes the response patterns of DJ123, we obtained transcriptome datasets from 48 leaf and 24 root samples across high- and low-P treatments from DJ123 and IR64. PCA analysis (Fig. 5) demonstrated that root- and leaf transcriptomes are tissue-specific explaining 89% variation, thus warranted a separate analysis. We focused our comparative analysis on the root tissues and between the HPC- and HPS-treatments. Because our PCA analysis showed that the 48 h P deficiency (HPS treatment) had induced transcriptional changes in the DJ123 root tissues that separate the transcriptome from that of the HPC (control) and towards the low-P treatments (Fig. 5C). Thus, the comparison between DJ123 and IR64 root transcriptome under HPS treatment might reveal the early events of low-P tolerance mechanisms in DJ123.

The GO enrichment analysis highlighted the genotypic differences between DJ123 and IR64 under low P conditions (Fig. 7). Unlike IR64, DJ123 root transcriptomes were specifically enriched with 14 P-starvation related GO terms under the HPS treatment (Fig. 7). This suggests that DJ123 employed different strategies to cope with low P supply. The DJ123-specific GO term ‘response to extracellular stimulus or nutrient levels’ could suggest that DJ123 responds to low P availability more strongly or earlier than IR64. The phosphatase and hydrolase GO terms suggest that DJ123 is more efficient in internal P scavenging activities to obtain more P from otherwise inaccessible sources^35,36^. The enrichment of the GO term ‘dephosphorylation’ may indicate activating P stress responsive mechanism^37^. The enrichment of these DJ123-specific GO terms suggests that DJ123 initiated several adaptive mechanisms in response to low P conditions.

We analysed the expression pattern of the genes that enriched the above GO terms in DJ123 under the HPS treatment. We compared this pattern between HPC and HPS treatments and between DJ123 and IR64 genotypes (Fig. 8). Our findings showed that the following genes were expressed to a greater extent in DJ123 in comparison to those in IR64 under the HPS treatment: (1) *Os01g0110100* (*PHO1;1*), which encodes a protein involved in transferring Pi from root to shoot^38^ and maintaining Pi homeostasis^39,40^, (2) *Os02g0168800* encodes a porphobilinogen deaminase^41^. Porphobilinogen deaminase has been reported to promote vegetative and reproductive development^42^, (3) *Os04g0326201* encodes a glycosyltransferase^43^. Glycosyltransferase is involved in synthesizing non-phosphorus lipid under phosphorus deprivation^44^, (4) *Os09g0506000* and *Os11g0151700* encode purple acid phosphatases^31,45^ and might play a role in Pi mobilization under low P condition^46^, (5) *Os11g0439100* encodes an oxidoreductase^47^ that is exuded by roots for degrading organic matter^48^, (6) *Os12g0189300* encodes a phosphoenolpyruvate carboxylase^47^. This enzyme, classified as an isomerase, is a major player for organic acid synthesis that is liberated in soil to scavenge Pi from insoluble P-complex^49^, (7) *Os02g0202200* (*SPX2*) is one of the six *SPX* genes in rice^50^. SPX2 modulates the activity of PHR2, the master protein that controls the expression of Phosphate Starvation Induced (PSI) genes in rice^50–52^. In summary, the above genes are reported in literature as P starvation inducible (PSI) genes. The greater expression of these PSI genes in DJ123 compared to that in IR64 under short-term P deprivation suggests that DJ123 strongly responds to P deprivation than IR64.

### Possible mechanisms for differential response patterns between DJ123 and IR64

Consistent with the change in tissue P concentration, DJ123 responded to a short-term P deprivation more drastically than IR64, in terms of the genome-wide gene expression pattern in root. Recent investigations revealed the mechanisms for induction of P starvation responses. Under P deficient conditions, the master regulators for P starvation response, PHR transcription factors, bind to the PHR1-binding sequence (P1BS) elements on the promoters of an array of PSI genes and induce their expression. On the other hand, under the P-replete conditions, SPX family proteins, including SPX2, bind to PHR transcription factors and inhibit their binding to P1BS elements or nuclear localization^52–55^. Since the interaction of SPX proteins and PHR proteins is mediated by inositol phosphate^28,56,57^, reduction in its concentration is a key for triggering of P starvation responses. Since the genes that are specifically induced in DJ123 in response to 48 h P deprivation are mostly PSI genes, it is likely that DJ123 more strongly or more promptly triggers P deficiency response pathways by reducing inositol phosphate and releasing PHR transcription factors upon short-term P deprivation compared with IR64. Alternatively, even though DJ123 maintains a relatively high P concentration in the whole root system, it is possible that the P concentration in specific root cells may be reduced, inducing low-P responsive genes in these cells. This is supported by the fact that the strengths of P deficiency responses are heterogenous among cells^58^. However, the potential for reduction in root inositol phosphate concentration or differential root cell P concentrations and consequently inducing PSI genes which may drive whole-plant responses to low P conditions certainly warrants further investigation.

## Conclusion

**Figure 9:**
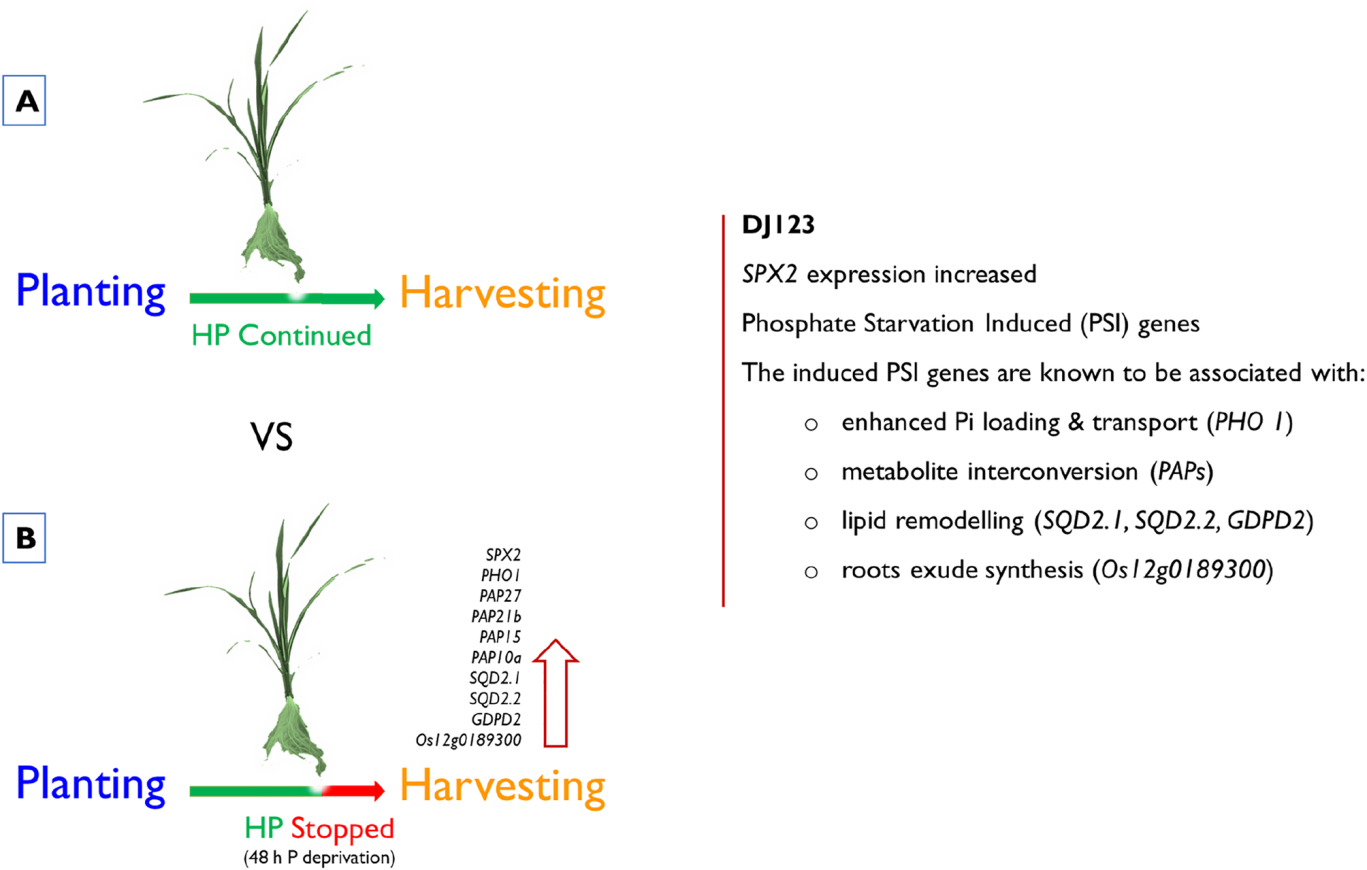
Diagram depicting the rapid response of DJ123 to low-P condition. High P and low-P conditions in the growing medium were represented by the green and red colors in the solid arrow, respectively. A. Plants were supplied with high P for the entire experimental period (42 days). B. High P supply was stopped after 40 days, then plants were harvested within 24–48 h. The response of DJ123 to low-P condition was determined by analyzing the differential gene expression in the root transcriptomes between A and B.

Considering together the entirety of our findings, our results demonstrate that DJ123 rapidly responded to the low-P availability in the growing medium, despite having abundant internal P to support growth and development. In response, DJ123 swiftly employed an array of the PSI genes. These PSI genes are reported in literature to be associated with low P adaptive mechanisms that included (1) increasing Pi uptake (*PHO1*), (2) scavenging P from metabolite interconversion (*PAPs*), (3) reducing P consumption by switching to non-phosphorus lipid synthesis (*SQD2.1*, *SQD2.2*), and (4) increasing organic acid synthesis that is liberated by roots for releasing P from the insoluble P-complexes (*Os12g0189300*) (Fig. 9). The ability to promptly deploy adaptive mechanisms in response to low-P in the growing media might underlie the remarkable low-P tolerance in DJ123. However, further investigations are required to determine what triggers these prompt responses in DJ123.

## Materials and methods

### Biological materials

Experimental research and field studies on plants complies with all relevant institutional, national, and international guidelines and legislation.

### Plant materials and treatment

Rice (*Oryza sativa* L.) genotypes DJ123 and IR64 were used in this study. DJ123 develops a larger root system earlier than IR64 under low-P conditions. This early root vigor allows DJ123 to explore a greater soil volume and improve P acquisition. In contrast, IR64 is a high-yielding modern cultivar but lacks early root vigor and is comparatively less tolerant to low P availability. DJ123 and IR64 seeds were washed with 70% ethanol once, followed by three washes with deionized water and incubated in petri dishes at 30 °C. Germinated seeds were transferred to mesh floating in a 6 L tray filled with deionized water. Nutrients except P and N were added in the tray as follows: 0.4 mL of 0.5 M K_2_SO_4_ and 1 M MgSO_4_·7H_2_O + 0.2 mL of 2000× Micronutrients (1× Micronutrients = 9 µM MnCl_2_·4H_2_O + 0.5 µM (NH_4_)6MO_7_O_24_·4H_2_O + 18.5 µM H_3_BO_3_ + 0.16 µM CuSO_4_·5H_2_O + 0.3 µM ZnSO_4_·7H_2_O) + 0.8 mL of 100 µM Ca + 1.12 mL of 10 µM Fe. pH in the trays was 5.4. Six days after germination (DAG), the trays were replenished with 0.2× Yoshida solution without P and N. The full-strength Yoshida solution (1×) is composed of: N, 2.86 mM (as NH_4_NO_3_); P, 0.05 mM (as NaH_2_PO_4_·2H_2_O); K, 1 mM (as K_2_SO_4_); Ca, 1 mM (as CaCl_2_); Mg, 1 mM (as MgSO_4_·7H_2_O); Mn, 9 μM as (MnCl_2_·4H_2_O); Mo, 0.5 μM (as (NH_4_)6MO_7_O_24_·4H_2_O); B, 18.5 μM (as H_3_BO_3_); Cu, 0.16 μM as (CuSO_4_·5H_2_O); Fe, 36 μM (as FeCl_3_·6H_2_O); Zn, 0.15 μM (as ZnSO_4_·7H_2_O)^59^. At 10 DAG, plants were transferred to 1.1 L black bottles containing low P (0.2 × Yoshida + 2 µM Na_2_HPO_4_) and high-P (0.2 × Yoshida + 50 µM Na_2_HPO_4_) solutions. Nutrient solutions in the bottles were completely replaced by fresh low- and high-P solution on every alternative day. Each bottle had two seedlings (technical replicates) and represented one biological replicate. Each treatment had three biological replicates. Plants were grown between September and November 2019, in a naturally lit temperature-controlled glass house at the Japan International Research Center for Agricultural Sciences, Tsukuba, Japan; at a mean temperature of 27 °C (23–33 °C) and a mean relative humidity of 40% (10–78%). Plants were regularly monitored for P-deficiency symptoms and harvested when the low-P treated plants appeared to be P-deficient. All low-P treatment replicates were harvested on DAG 41 and the high-P treatment ones on DAG 42. For transcriptomic study, first and third youngest leaves and roots were collected from both genotypes of all treatments and frozen immediately in liquid nitrogen and stored at − 80 °C until RNA extraction.

### Phosphorus concentration and content

An aliquot of approximately 100 mg dried sample was pre-digested in 8 mL of a mixture of HNO3, HClO4 (3:1) in 50 mL of acid digestion tubes overnight, followed by digestion at 105 °C for 1 h and removal of cHNO3 at 140˚C for 1 h in a block digester. The block digester was heated up to a maximum of 170˚C to dehydrate any silica present in the digest. After cooling, the digested solution was diluted with DI water to a volume of 50 mL. The digested solution was centrifuged at 12,000 rpm for 5 min and the supernatant was used for P concentration measurement using the molybdenum blue method^60^. The P content in leaves and roots was calculated as P concentration in the corresponding organ times its dry biomass.

### RNA extraction

Total RNA was extracted from rice leaf and root tissues using TRIzol Reagent (Invitrogen, Carlsbad, CA, USA) according to the manufacturer’s instructions including DNase treatment. The quantity and purity of the total RNA in each sample were determined using a NanoDrop spectrophotometer (Thermo Scientific). The RNA integrity was checked using an Agilent Technologies 2100 Bioanalyzer (Agilent Technologies, Inc., Santa Clara, CA, USA). The samples used for sequencing had a RIN value ranging from 6.2 to 8.6.

### cDNA library construction and illumina sequencing

The cDNA libraries were individually prepared from each sample using the “TruSeq Stranded mRNA Low Throughput (LT) Sample Preparation Kit (Illumina, San Diego, CA, USA)” following the manufacturer’s instructions (TruSeq Stranded mRNA Sample Preparation Guide, Part # 15031047 Rev. E). The DNA libraries were purified from the PCR reactions using AMPure XP Beads, and quantified using Agilent Technologies 2100 Bioanalyzer. The sequencing libraries were sequenced on an Illumina NovaSeq6000 sequencing platform by Macrogen Japan (Kyoto, Japan).

### Quality control

The quality control and reference mapping of the RNA-Seq data were carried out using open-source tools in Ubuntu command-line interface. First, the quality of the raw reads was evaluated using FastQC (http://www.bioinformatics.babraham.ac.uk/projects/fastqc/) commands. Then, the raw reads were processed using ‘trimmomatic’ package for trimming the Illumina PE adapters (TruSeq3-PE-2.fa:2:30:10), removing the low-quality or poly-N bases from the ends that were below quality score 3, removing the sequences when the average quality per base dropped below 15 in a 4-base sliding window, and dropping the reads with less than 25 bases in length. This led to a set of clean and high-quality RNA-seq reads for subsequent analyses.

### Reference mapping

Rice reference genome (fasta) and genome annotation (GTF) files were downloaded from ‘EnsemblPlants’ website (*Oryza sativa* Japonica Group, https://plants.ensembl.org/info/data/ftp/index.html). The pre-processed paired-end RNA-Seq reads were mapped to the reference genome using HISAT2^61^. First, the splice and exon information from the rice GTF file were extracted using two python scripts—‘extract_splice_sites.py’ and ‘extract_exons.py’—provided with the HISAT2 package (https://cloud.biohpc.swmed.edu/index.php/s/hisat2-220-source/download). Then, this information together with the reference genome in ‘fasta’ format were used as input to build a reference genome index followed by aligning the reads to these indexes. The alignment was produced in ‘sam’ format. ‘samtools sort’ function was used to convert the ‘sam’ format to ‘bam’, and sort the bam files^62^. The sorted bam files were passed to StringTie^63^ for assembling transcripts and quantifying expressed genes and transcripts. However, some samples might have partial read coverage for some transcripts resulting in partial assembly of these transcripts. The ‘StringTie merge’ function was used to merge all the gene structures found in any of the samples and re-estimate the transcript abundances using the merged structures. This created a consistent set of transcripts across the samples making them comparable in the downstream analysis.

StringTie uses MSTRG gene ids. We ran a Python postprocessing script, ‘mstrg_prep.py’ (Pertea, https://gist.github.com/gpertea/4207fa9cb30fe7fec0eb52bd29b9a976) that appends reference gene ids to the MSTRG gene ids used in StringTie. However, we used ‘stringtie-e-B’ function to estimate the transcript abundance and create count tables for differentially expressed genes. We processed the row count table using a Python script ‘prepDE.py’ (Pertea M, http://ccb.jhu.edu/software/stringtie/index.shtml?t=manual#de) for performing differential gene expression analysis using the DESeq2 package.

### Differential gene expression analysis

We used DESeq2 R package for analyzing the differentially expressed genes (DEGs) using the ‘design =  ~ Genotypes + Treatments + Genotypes:Treatments’ Generalized Linear Model^64^. We filtered out the genes with less than 10 counts. The ‘relevel’ function was used to manually set ‘IR64’ and ‘HPC’ as a reference for genotype and treatment, respectively. By default, the ‘DESeq’ function normalized the raw read counts for library size, estimated the dispersion of counts for each DEG, and calculated the significance of coefficients using ‘nbinomWaldTest’. Then, we estimated the treatment effects on the DEGs using the ‘results’ function with a threshold for False Discovery Rate set at 0.005. We used ‘lfcShrink’ function to perform shrinkage on log2 fold change. All ‘log2 fold change’ values presented in this manuscript are shrinkage estimated value. The thresholds to define significantly DEGs across the genotypes, treatments and tissues were ‘padj < 0.001’ and ‘log2FoldChange < −1|log2FoldChange > 1’. To determine the global pattern of the DEGs in the samples, we performed Principal Components Analysis (PCA) on expression values using ‘plotPCA’. Using ‘EnhancedVolcano’^65^, we determined the DEGs that were treatment- or genotype-specific.

### Gene Ontology (GO) term enrichment and pathway analysis

We refined a list of DEGs in DJ123 roots compared with IR64 under low-P treatment. We functionally annotated these DEGs against a series of ‘Annotation Data Set’ viz. ‘GO biological process complete’, ‘GO molecular function complete’, ‘GO cellular component complete’, ‘PANTHER Pathways’, ‘PANTHER GO-Slim Biological Process’, ‘PANTHER GO-Slim Molecular Function’, ‘PANTHER GO-Slim Cellular Component’ and ‘PANTHER Protein Class’ that are implemented in PANTHER classification system (http://pantherdb.org/tools/compareToRefList.jsp or http://www.pantherdb.org)^66^. We used ‘*Oryza sativa* all genes in the database’ as a reference list. The annotation used ‘Fisher’s Exact’ test with ‘No correction’.

### Validation of the RNA-seq data

We validated our RNA-seq data using quantitative real-time PCR (qRT-PCR) assay on a CFX96 Touch Real-Time PCR system (BioRad, USA). The qRT-PCR assay was carried out on a panel of eight genes including two house-keeping ones, ELF1 (Elongation factor) and UBI (Ubiquitin) (Supplementary Table S3). Gene specific primers were designed using the Primer3 software keeping the default parameters^67^. Total RNA from three independent biological replicates was reverse transcribed (RT) into cDNA using the PrimeScript RT Enzyme Mix I (Takara, Japan). qRT-PCR reaction volume was 10μL containing 5 µL TB Green^®^ Premix Ex Taq™ II (Takara, Japan), 1 µL cDNA, 0.04 µL of each of the primers (100 μM) and 3.92 μL RNase-free water.

qRT-PCR cycling conditions were as follows: 95 °C for 30 s, followed by 40 cycles at 95 °C for 5 s, 60 °C for 30 s. For melt curve analysis, the denaturation temperature was incrementally increased from 65.0 to 95.0 °C by 0.5 °C per cycle. Relative gene expression level was calculated using the standard-curve method and expressed as fold-change.

## Supplementary Information

Supplementary Information.

## Data Availability

RNA-seq data generated in the study have been deposited in the National Centre for Biotechnology Information (NCBI) under BioProject ID PRJNA823747, BioSample ID SUB11348183 and Sequence Read Archive (SRA) submission ID SUB11348231.
