# Supplementary material for: Comparative transcriptome analysis reveals a rapid response to phosphorus deficiency in a phosphorus-efficient rice genotype
Source: Sci Rep. 2022 Jun 8;12:9460. doi: 10.1038/s41598-022-13709-w (PMC9177723; doi:10.1038/s41598-022-13709-w)
Supplement: Supplementary file 1 — Supplementary Information. [file 41598_2022_13709_MOESM1_ESM.pdf]

Comparative transcriptome analysis reveals a rapid response to phosphorus deficiency in a phosphorus-efficient rice genotype

M. Asaduzzaman Prodhan[1,2,*], Juan Pariasca-Tanaka[1], Yoshiaki Ueda[1], Patrick E. Hayes[1,2], Matthias Wissuwa[1]

[1]Crop, Livestock and Environment Division, Japan International Research Center for Agricultural Sciences, Tsukuba, Ibaraki, Japan; [2]School of Biological Sciences, The University of Western Australia, 35 Stirling Highway, Perth, WA 6009, Australia

*Correspondence: asad.prodhan@uwa.edu.au

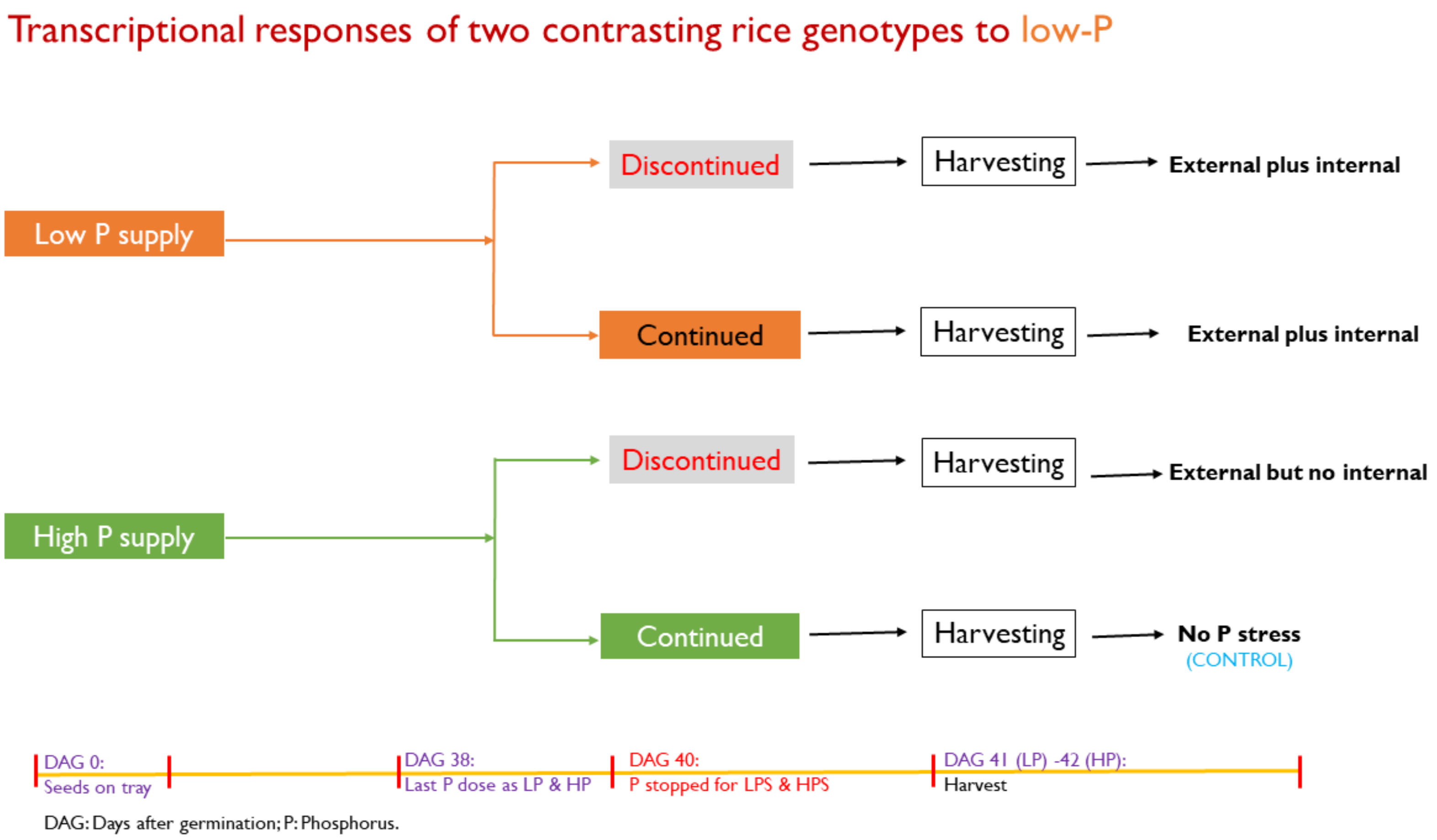

Supplementary Fig S1. Experimental Layout

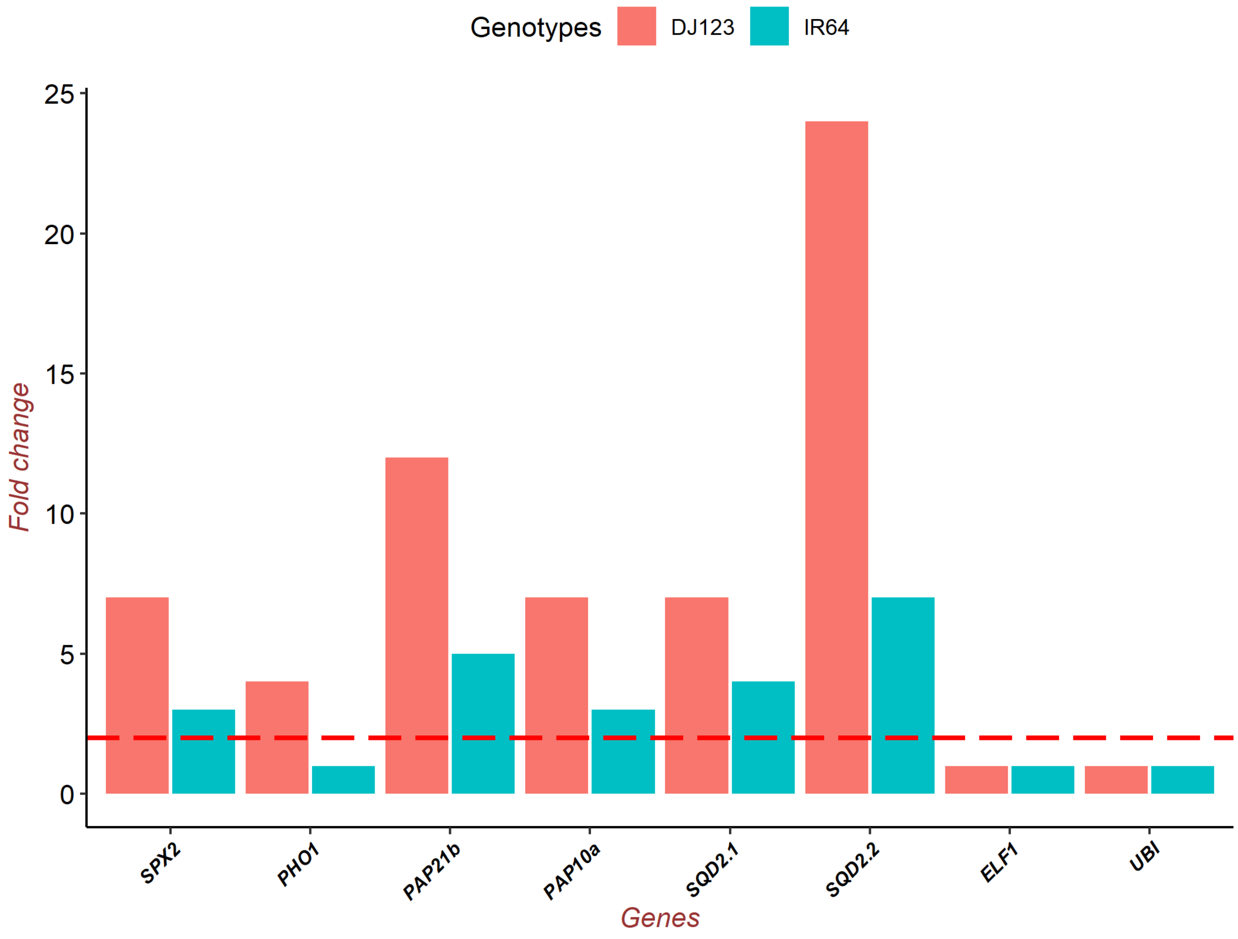

Supplementary Fig S2. Validation of the differentially expressed genes in the transcriptome analysis using qPCR assay. 'Fold change' was calculated in the same way like in the transcriptome analysis, i.e., the transcript abundance under HPS treatment was divided by that under the HPC treatment in each genotype. The red line represents two-fold up-regulation of the corresponding gene in HPS treatment compared to that in HPC.

Supplementary Table S1. Differentially expressed genes (DEGs) and enriched GO terms in DJ123 and IR64 root samples by short-time P deprivation.

Treatments	Genotypes	Directions	Categories	DEG No	Fold_enrichment	pValue	Mapped_ID
HPS	Both	Up	Protein	9	4	0.000278682	['Os01g0142300', 'Os07g0100300', 'Os01g0776600', 'Os09g0321200', 'Os08g0299400', 'Os07g0668700', 'Os11g0658900', 'Os12g0633800', 'Os02g0514500']
HPS	Both	Up	MF	2	1265	2.65E-06	['Os01g0142300', 'Os07g0100300']
HPS	Both	Up	BP	2	759	5.56E-06	['Os01g0142300', 'Os07g0100300']
HPS	Both	Up	BP	2	759	5.56E-06	['Os01g0142300', 'Os07g0100300']
HPS	Both	Up	BP	5	15	1.94E-05	['Os01g0142300', 'Os07g0100300', 'Os09g0321200', 'Os08g0299400', 'Os02g0514500']
HPS	Both	Up	BP	7	15	2.87E-07	['Os01g0142300', 'Os07g0100300', 'Os09g0321200', 'Os08g0299400', 'Os07g0668700', 'Os11g0658900', 'Os02g0514500']
HPS	Both	Up	BP	3	102	4.07E-06	['Os01g0142300', 'Os07g0100300', 'Os08g0299400']
HPS	Both	Up	BP	3	63	1.66E-05	['Os01g0142300', 'Os07g0100300', 'Os08g0299400']
HPS	Both	Up	BP	3	52	2.87E-05	['Os01g0142300', 'Os07g0100300', 'Os08g0299400']
HPS	Both	Up	BP	3	95	4.96E-06	['Os01g0142300', 'Os07g0100300', 'Os08g0299400']
HPS	Both	Up	BP	3	95	4.96E-06	['Os01g0142300', 'Os07g0100300', 'Os08g0299400']
HPS	Both	Up	BP	3	42	5.22E-05	['Os09g0321200', 'Os11g0658900', 'Os02g0514500']
HPS	IR	Down	Protein	6	7	0.000225539	['Os04g0530600', 'Os04g0624450', 'Os08g0465700', 'Os01g0263300', 'Os10g0419400', 'Os01g0543100']

Treatments	Genotypes	Directions	Categories	DEG No	Fold_enrichment	pValue	Mapped_ID
HPS	IR	Up	MF	3	26	0.000204982	['Os12g0193200', 'Os03g0661300', 'Os06g0114200']
HPS	IR	Up	MF	8	5	6.65E-05	['Os06g0192600', 'Os05g0514200', 'Os12g0193200', 'Os05g0440800', 'Os12g0197100', 'Os03g0661300', 'Os03g0282100', 'Os06g0114200']
HPS	IR	Up	MF	8	5	0.000179336	['Os06g0192600', 'Os05g0514200', 'Os12g0193200', 'Os05g0440800', 'Os12g0197100', 'Os03g0661300', 'Os03g0282100', 'Os06g0114200']
HPS	IR	Up	MF	8	4	0.000263413	['Os06g0192600', 'Os05g0514200', 'Os12g0193200', 'Os05g0440800', 'Os12g0197100', 'Os03g0661300', 'Os03g0282100', 'Os06g0114200']
HPS	IR	Up	MF	3	26	0.000204982	['Os12g0193200', 'Os03g0661300', 'Os06g0114200']
HPS	IR	Up	MF	3	26	0.000217726	['Os12g0193200', 'Os03g0661300', 'Os06g0114200']
HPS	IR	Up	MF	8	5	9.42E-05	['Os06g0192600', 'Os05g0514200', 'Os12g0193200', 'Os05g0440800', 'Os12g0197100', 'Os03g0661300', 'Os03g0282100', 'Os06g0114200']
HPS	IR	Up	MF	8	5	0.000179336	['Os06g0192600', 'Os05g0514200', 'Os12g0193200', 'Os05g0440800', 'Os12g0197100', 'Os03g0661300', 'Os03g0282100', 'Os06g0114200']
HPS	IR	Up	MF	8	4	0.00027347	['Os06g0192600', 'Os05g0514200', 'Os12g0193200', 'Os05g0440800', 'Os12g0197100', 'Os03g0661300', 'Os03g0282100', 'Os06g0114200']
HPS	IR	Up	MF	8	5	9.07E-05	['Os06g0192600', 'Os05g0514200', 'Os12g0193200', 'Os05g0440800', 'Os12g0197100', 'Os03g0661300', 'Os03g0282100', 'Os06g0114200']

Treatments	Genotypes	Directions	Categories	DEG No	Fold_enrichment	pValue	Mapped_ID
HPS	IR	Up	MF	8	5	9.83E-05	['Os06g0192600', 'Os05g0514200', 'Os12g0193200', 'Os05g0440800', 'Os12g0197100', 'Os03g0661300', 'Os03g0282100', 'Os06g0114200']
HPS	IR	Up	MF	8	5	0.000103782	['Os06g0192600', 'Os05g0514200', 'Os12g0193200', 'Os05g0440800', 'Os12g0197100', 'Os03g0661300', 'Os03g0282100', 'Os06g0114200']
HPS	IR	Up	MF	3	26	0.000204982	['Os12g0193200', 'Os03g0661300', 'Os06g0114200']
HPS	IR	Up	MF	3	26	0.000204982	['Os12g0193200', 'Os03g0661300', 'Os06g0114200']
HPS	IR	Up	MF	3	26	0.000220335	['Os12g0193200', 'Os03g0661300', 'Os06g0114200']
HPS	IR	Up	MF	3	26	0.000220335	['Os12g0193200', 'Os03g0661300', 'Os06g0114200']
HPS	DJ	Up	Protein	5	17	1.54E-05	['Os11g0434000', 'Os03g0848200', 'Os09g0506000', 'Os11g0151700', 'Os07g0558200']
HPS	DJ	Up	Protein	18	2	0.000297858	['Os12g0189300', 'Os08g0191700', 'Os03g0848200', 'Os06g0204400', 'Os09g0506000', 'Os05g0387200', 'Os11g0151700', 'Os07g0558200', 'Os04g0326201', 'Os02g0168800', 'Os03g0184300', 'Os11g0434000', 'Os07g0638600', 'Os04g0107600', 'Os11g0210300', 'Os11g0439100', 'Os01g0847600', 'Os09g0513100']
HPS	DJ	Up	MF	4	59	9.56E-07	['Os11g0434000', 'Os03g0848200', 'Os09g0506000', 'Os11g0151700']
HPS	DJ	Up	MF	7	11	3.15E-06	['Os01g0855000', 'Os11g0434000', 'Os03g0848200', 'Os11g0615000', 'Os09g0506000', 'Os11g0151700', 'Os07g0558200']

Treatments	Genotypes	Directions	Categories	DEG No	Fold_enrichment	pValue	Mapped_ID
HPS	DJ	Up	MF	7	10	8.72E-06	['Os01g0855000', 'Os11g0434000', 'Os03g0848200', 'Os11g0615000', 'Os09g0506000', 'Os11g0151700', 'Os07g0558200']
HPS	DJ	Up	BP	4	135	4.83E-08	['Os10g0392600', 'Os01g0110100', 'Os05g0387200', 'Os02g0202200']
HPS	DJ	Up	BP	4	49	2.01E-06	['Os10g0392600', 'Os01g0110100', 'Os05g0387200', 'Os02g0202200']
HPS	DJ	Up	BP	4	45	2.68E-06	['Os10g0392600', 'Os01g0110100', 'Os05g0387200', 'Os02g0202200']
HPS	DJ	Up	BP	4	35	6.74E-06	['Os10g0392600', 'Os01g0110100', 'Os05g0387200', 'Os02g0202200']
HPS	DJ	Up	BP	4	31	1.07E-05	['Os10g0392600', 'Os01g0110100', 'Os05g0387200', 'Os02g0202200']
HPS	DJ	Up	BP	4	39	4.51E-06	['Os10g0392600', 'Os01g0110100', 'Os05g0387200', 'Os02g0202200']
HPS	DJ	Up	BP	4	39	4.79E-06	['Os10g0392600', 'Os01g0110100', 'Os05g0387200', 'Os02g0202200']
HPS	DJ	Up	BP	4	40	4.24E-06	['Os10g0392600', 'Os01g0110100', 'Os05g0387200', 'Os02g0202200']
HPS	DJ	Up	BP	7	11	3.74E-06	['Os01g0855000', 'Os11g0434000', 'Os03g0848200', 'Os11g0615000', 'Os09g0506000', 'Os11g0151700', 'Os07g0558200']

Supplementary Table S2. Differentially expressed genes (DEGs) in DJ123- and IR64-specific GO terms.

Genes	Genotypes	Transcript_Abundance_HPS_by_HPC
Os07g0638600|prx105	DJ	0.6
Os01g0855000|GPAT	DJ	0.6
Os04g0107600|ADC2	DJ	0.8
Os02g0168800|PBD	DJ	0.8
Os08g0191700|GLYI-11	DJ	1.3
Os11g0210300|ADH1	DJ	1.4
Os01g0847600|ARK1	DJ	1.5
Os03g0184300	DJ	2.3
Os11g0434000	DJ	2.5
Os11g0434000	DJ	2.5
Os11g0615000	DJ	2.5
Os06g0204400	DJ	2.5
Os01g0110100|Pho1	DJ	2.6
Os07g0558200	DJ	2.7
Os03g0848200	DJ	2.7
Os05g0387200	DJ	3
Os09g0513100	DJ	3
Os01g0855000	DJ	3.7
Os02g0202200|SPX2	DJ	4.7
Os02g0202200	DJ	6.8
Os11g0151700|PAP21b	DJ	7.7
Os09g0506000|PAP27A	DJ	8.6
Os04g0326201	DJ	9.3
Os02g0168800	DJ	9.5
Os11g0439100	DJ	9.8
Os01g0110100	DJ	22.1
Os12g0189300	DJ	30.7
Os11g0151700	DJ	64.8
Os09g0506000	DJ	265
Os07g0638600|prx105	IR	0.2
Os01g0847600	IR	0.3
Os04g0107600|ADC2	IR	0.6
Os04g0326201	IR	0.9
Os10g0392600	IR	1
Os08g0191700|GLYI-11	IR	1
Os02g0168800|PBD	IR	1
Os01g0847600|ARK1	IR	1.1
Os02g0168800	IR	1.1
Os01g0855000|GPAT	IR	1.1
Os09g0513100	IR	1.3
Os11g0210300|ADH1	IR	1.4
Os03g0184300	IR	1.4
Os01g0110100|Pho1	IR	1.5
Os06g0204400	IR	1.5
Os11g0439100	IR	1.5

Genes	Genotypes	Transcript_Abundance_HPS_by_HPC
Os11g0151700|PAP21b	IR	1.6
Os11g0434000	IR	1.6
Os11g0434000	IR	1.6
Os03g0848200	IR	1.7
Os11g0615000	IR	1.9
Os01g0855000	IR	1.9
Os05g0387200	IR	2
Os07g0558200	IR	2
Os02g0202200|SPX2	IR	2.1
Os01g0110100	IR	2.3
Os11g0210300	IR	2.5
Os09g0506000|PAP27A	IR	3.4
Os02g0202200	IR	3.5
Os12g0189300	IR	4.2
Os09g0506000	IR	4.6
Os08g0191700	IR	13
Os11g0151700	IR	27.3
Os07g0638600	IR	144.8

Supplementary Table S3. The genes and primers for qPCR assay

Name	Rap_IDs	Forward (F)_Primer	F_Primer_Len	Riverse (R)_Primer	R_Primer_Len
SQD2.2	Os01g0142300	ACTGGAATGATGCAAGGCGA	20	TCTCCTGGGGTGAACAGGAA	20
SQD2.1	Os07g0100300	CAGAAAGCTTCCATCCCCGT	20	TGAATGGCCCATCACCAACA	20
PHO1	Os01g0110100	CAGCTGCATGCGTATTGCTC	20	CCACAAAGGCATGCTTCCAC	20
SPX2	Os02g0202200	CGGGGAGGTGAAAACGAGAA	20	GCAGGCGAAACAACGATACC	20
PAP21b	Os11g0151700	GGAAGGCCTTGCTCTCAAGT	20	AAGCTCGCCTCCCTGAACTC	20
PAP10a	Os01g0776600	GTGGTTCTTTCTCCGCTCCA	20	AACGCCACGAAACCAATGTG	20
ELF1	Os07g0631100	AAGAGGAAGTCAGCGGCTAAG	21	CAGAATGGGCAGGAAAATACA	21
UBI	Os02g0261100	CTCAAGGACCTGCAGAAGGA	20	ATGGACCCATCAGTGTTGC	19
